# Complete Genome Sequences of Two Swiss Hepatitis E Virus Isolates from Human Stool and Raw Pork Sausage

**DOI:** 10.1128/genomeA.00888-17

**Published:** 2017-08-31

**Authors:** Jakub Kubacki, Cornel Fraefel, Marco Jermini, Petra Giannini, Gladys Martinetti, Paolo Ripellino, Enos Bernasconi, Xaver Sidler, Roger Stephan, Claudia Bachofen

**Affiliations:** a Institute of Virology, Vetsuisse Faculty University of Zurich, Zurich, Switzerland; b Department of Health, Cantonal Laboratory Ticino, Division of Public Health, Bellinzona, Switzerland; c Department of Laboratory Medicine, Laboratory of Bacteriology, Ente Ospedaliero Cantonale, Bellinzona, Switzerland; d Neurology Department, Neurocenter of Southern Switzerland, Ente Ospedaliero Cantonale, Bellinzona, Switzerland; e Internal Medicine and Infectious Diseases Department, Ente Ospedaliero Cantonale, Bellinzona, Switzerland; f Department of Farm Animals, Division of Swine Medicine, Vetsuisse Faculty University of Zurich, Zurich, Switzerland; g Institute for Food Safety and Hygiene, Vetsuisse Faculty University of Zurich, Zurich, Switzerland

## Abstract

We present here the full-length genome sequences of two hepatitis E virus genotype 3 (HEV-3) isolates from a human stool sample from a patient with acute hepatitis and a raw sausage containing pig liver. Sequence analysis implies that Swiss HEV isolates may form a novel subgroup of HEV-3 viruses.

## GENOME ANNOUNCEMENT

Hepatitis E virus (HEV) is a major cause of acute hepatitis worldwide. It is a small nonenveloped or quasienveloped virus (1) with a positive-sense single-stranded RNA genome of approximately 7.2 kb and belongs to the family *Hepeviridae*. Genotypes 1 and 2 (HEV-1 and HEV-2, respectively) circulate within the human population and are a major health issue in developing countries. HEV-3 and HEV-4 are zoonotic viruses that are highly prevalent in porcine species and may be transmitted to humans by the consumption of pig liver and meat (2). HEV is also present in Switzerland (3, 4); however, aside from a single full-length sequence that was published recently (5), no further information on the diversity of HEV strains is available.

Here, we present the full-genome sequences of two HEV isolates from a human and a food sample. The stool sample originated in a 78-year-old male hospitalized in October 2016 in Lugano (Canton Ticino, southern Switzerland) with acute hepatitis and was collected within 10 days from symptom onset. Total RNA was extracted using the QIAamp viral RNA minikit, according to the manufacturer’s instructions (Qiagen GmbH, Germany). The food sample was a traditional raw dry-cured pork sausage containing pork liver (“mortadella di fegato crudo”), sold by a local butcher shop. For the RNA extraction, a combination of TRI reagent (Lucerna-Chem AG, Luzern, Switzerland) and the NucliSENS easyMAG system (bioMérieux, Geneva, Switzerland) was used. Both RNAs were shown to be HEV positive by a commercial quantitative real-time reverse transcription-PCR (RT-PCR) (Ceeramtools [bioMérieux, Geneva, Switzerland]). To prepare the RNA samples for next-generation sequencing (NGS), sequence-independent single-primer amplification was performed (6), and the purified amplicons were used for the construction of sequencing libraries using the NEBNext Ultra II library preparation kit (BioConcept, Allschwil, Switzerland). A paired-end NGS run of 2 × 150 nucleotide read length was performed at the Functional Genomic Center Zurich using the Illumina NextSeq 500 machine. Alignment of the reads to full-length hepatitis E virus genomes using the SeqMan NGen software (DNAStar [Lasergene, Madison, WI, USA]) revealed best match to the recently published Swiss HEV-3 strain SW/16-0282 (GenBank accession no. KY780957 [5]). The consensus sequences of the alignments were near full length, with only 39 and 69 nucleotide gaps in the open reading frame 2 (ORF2) region for the fecal sample and the sausage sample, respectively. The gaps were bridged by Sanger sequencing using specific primers binding upstream and downstream of the gaps. The 7,222-nucleotide full-length sequences [excluding the poly(A) tail] contain the 3 known HEV ORFs that are specified in detail in the GenBank entries. The sequences are identical except for 21 positions with nucleotide ambiguities that were confirmed by Sanger sequencing and point to quasispecies diversity. It is very likely that the two isolates belong to the same virus strain. Interestingly, this strain shows 95% identity to the only other fully sequenced Swiss isolate (accession no. KY780957 [5]) but only 88% identity to other HEV-3 strains. Analysis of more Swiss HEVs is necessary to confirm the existence of a Swiss-specific HEV-3 subcluster.

### Accession number(s).

Both sequences are deposited in GenBank under the accession numbers MF346772 and MF346773.
